# The Role of Ferroptosis and Cuproptosis in Tuberculosis Pathogenesis: Implications for Therapeutic Strategies

**DOI:** 10.3390/cimb47020099

**Published:** 2025-02-05

**Authors:** John Dawi, Stephen Affa, Kevin Kafaja, Yura Misakyan, Samuel Kades, Surbi Dayal, Sabrina Fardeheb, Ananya Narasimhan, Kevin Tumanyan, Vishwanath Venketaraman

**Affiliations:** 1 College of Osteopathic Medicine of the Pacific, Western University of Health Sciences, Pomona, CA 91766, USA; john.dawi@westernu.edu (J.D.); kevin.kafaja@westernu.edu (K.K.); yura.misakyan@westernu.edu (Y.M.); samuel.kades@westernu.edu (S.K.); surbi.dayal@westernu.edu (S.D.); sabrina.fardeheb@westernu.edu (S.F.); ananya.narasimhan@westernu.edu (A.N.); 2 Department of Chemistry, Physics, and Engineering, Los Angeles Valley College, Valley Glen, CA 91401, USA; affasn7875@student.laccd.edu; 3 College of Podiatric Medicine, Western University of Health Sciences, Pomona, CA 91766, USA; kevin.tumanyan@westernu.edu

**Keywords:** ferroptosis, cuproptosis, tuberculosis, oxidative stress, *M.tb*, macrophage

## Abstract

Tuberculosis (TB) caused by *Mycobacterium tuberculosis* (*M.tb*) remains a global health crisis, with over 10 million people affected annually. Despite advancements in treatment, *M.tb* has developed mechanisms to evade host immune responses, complicating efforts to eradicate the disease. Two emerging cell death pathways, ferroptosis and cuproptosis, have been linked to TB pathogenesis. Ferroptosis, an iron-dependent form of cell death, is driven by lipid peroxidation and reactive oxygen species (ROS) accumulation. This process can limit *M.tb* replication by depleting intracellular iron and inducing macrophage necrosis. However, excessive ferroptosis may lead to tissue damage and aid bacterial dissemination. Cuproptosis, triggered by copper accumulation, disrupts mitochondrial metabolism, leading to protein aggregation and cell death. *M.tb* exploits both iron and copper metabolism to survive within macrophages, manipulating these processes to resist oxidative stress and immune responses. This review examines the roles of ferroptosis and cuproptosis in TB, discussing how *M.tb* manipulates these pathways for survival. While therapeutic strategies targeting these processes, such as ferroptosis inducers (Erastin, RSL3) and inhibitors (Ferrostatin-1) and copper ionophores (Disulfiram, Elesclomol) and chelators, show promise, the limited understanding of these pathways and potential off-target effects remains a significant challenge. Further exploration of these pathways may provide insights into the development of targeted therapies aimed at controlling *M.tb* infection while minimizing host tissue damage. By elucidating the complex interactions between ferroptosis, cuproptosis, and TB, future therapies could better address bacterial resistance and improve clinical outcomes.

## 1. Introduction

In 2022, tuberculosis (TB) was identified by the World Health Organization (WHO) as the second leading cause of death from a single infectious agent, following COVID-19. It accounted for nearly twice as many deaths as HIV/AIDS. Despite being preventable and treatable, TB infected over 10.6 million people globally in 2022, resulting in approximately 1.3 million deaths [1]. In the United States, TB cases rose by 16% from 8320 cases in 2022 to 9615 cases in 2023 [2]. Given the severity and rising incidence of TB, the United Nations (UN) and the WHO have set an ambitious goal to end the global TB epidemic by 2030. Tuberculosis is an infectious disease caused by *M.tb*, primarily transmitted via airborne particles from infected individuals. Although TB predominantly affects the lungs, it can spread to other parts of the body. The disease is often linked to poor sanitation, inadequate hygiene, poverty, and the ease of transmission between people [3]. Diagnostic protocols for TB rely on acid-fast bacilli (AFB) testing and *M.tb* cultures as first-line and gold-standard methods [4]. TB diagnosis is based on several criteria: (1) a positive tuberculin skin test, (2) abnormal chest radiographs showing Ghon complexes (hilar lymphadenopathy and peripheral granulomatous lesions in the middle or lower lung lobes), and (3) clinical symptoms such as low-grade fever, night sweats, malaise, fatigue, weight loss, hemoptysis, and chronic productive cough [5,6]. The potential for diagnostic errors due to contamination and clerical mistakes makes TB diagnosis challenging.

Patients with TB are categorized as either having latent tuberculosis infection (LTBI)—an asymptomatic, non-infectious state—or active TB disease, which is infectious and symptomatic. LTBI is characterized by bacteria in a dormant state without clinical symptoms [4]. However, immunocompromised individuals or those untreated for LTBI are at increased risk of progressing to active TB. Active TB involves bacterial proliferation and symptomatic manifestations, such as fever, fatigue, weight loss, and chest pain [3]. Individuals with active TB are contagious and capable of transmitting the bacteria to others. The pathogenesis of active TB involves the spread of *M.tb* between infected macrophages, a process influenced by host cell death pathways. Studies indicate that *M.tb*-infected macrophages undergo apoptosis, encapsulating intracellular bacteria within apoptotic bodies. These bodies are subsequently phagocytosed by uninfected macrophages, limiting *M.tb* replication [7]. However, necrotic death of *M.tb*-infected macrophages can promote bacterial dissemination due to extracellular bacilli release [8,9]. This underscores the need for deeper investigation into regulated cell death’s role in TB pathogenesis.

**Figure 1:**
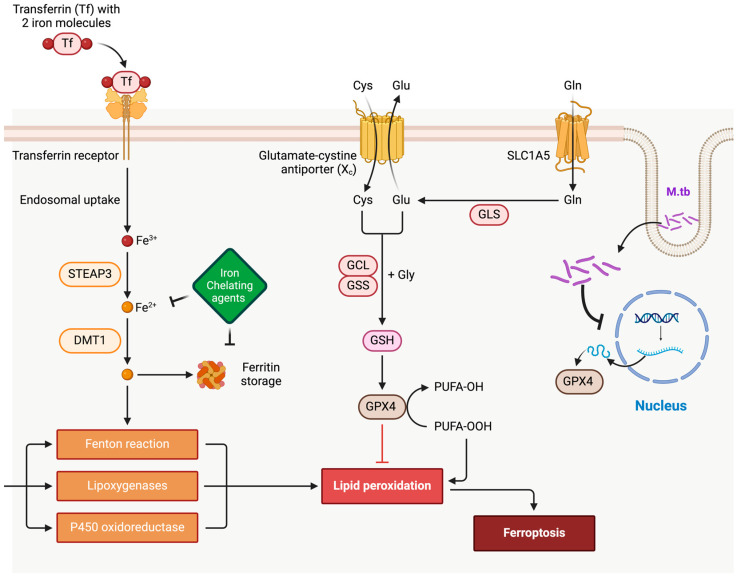
The process of ferroptosis. The overview of ferroptosis, as depicted in the accompanying figure, highlights its essential components. It begins with the metabolic generation of ROS, serving as key substrates for phospholipid peroxidation. The figure above portrays iron-dependent reactions crucial for initiating and propagating phospholipid peroxidation, integral to ferroptosis progression. It also emphasizes the role of surveillance mechanisms, particularly GPX4, in regulating and containing phospholipid peroxidation within this complex biological pathway. Furthermore, the figure demonstrates the inhibitory influence of GSH and iron chelating agents on iron-dependent reactive oxygen species (ROS) generation. The SLC1A5 transporter regulates the rate-limiting step of glutamine uptake, which is catalyzed and broken down into glutamate through GLS (glutaminase) as a part of GSH generation. STEAP3 and DMT1 are endosomal proteins. The complex of transferrin with Tfr1 is internalized into the endosomal compartment. Iron is released from the complex with transferrin inside endosomes, and then, iron is transported to the cytosol by DMT1. Figure created with BioRender.com, accessed on 25 October 2024. STEAP3: Six-Transmembrane Epithelial Antigen of Prostate 3. DMT1: Divalent Metal Transporter 1. GLS: Glutaminase. GSS: Glutathione Synthetase. GPX4: Glutathione Peroxidase 4. GSH: Glutathione. SLC1A5: Solute Carrier Family 1 Member. P450: Cytochrome P450.

One regulated cell death mechanism involved in TB pathogenesis is ferroptosis, exhibiting characteristics of both apoptosis and necrosis (Figure 1). Ferroptosis results from iron overload, which generates lipid peroxides in cell membranes. Normally, glutathione peroxidase 4 (Gpx4) detoxifies lipid peroxides through glutathione (GSH) oxidation. However, Gpx4 activity is impaired by glutathione depletion or direct inhibition by ferroptosis inducers, such as RSL3. Iron overload exacerbates lipid peroxidation but does not directly inhibit Gpx4. This process destabilizes the plasma membrane due to elevated iron and lipid peroxidation, decreased GSH, and reduced Gpx4 activity [9,10]. Notably, polyunsaturated fatty acids (PUFAs) in phospholipids are key substrates for lipid peroxidation, driving ferroptosis-associated membrane damage. Research by Amaral et al. hypothesized that ferroptosis may play a key role in *M.tb* infections. The rationale includes the role of iron in enhancing *M.tb* infection risk and disease progression. Elevated iron levels have been associated with increased susceptibility to active TB [11]. Further, *M.tb*-infected macrophages exhibit iron accumulation, lipid peroxidation, and low Gpx4 expression, resulting in cell necrosis [12]. Inhibiting this process using the ferroptosis inhibitor ferrostatin-1 and the iron chelator pyridoxal isonicotinoyl hydrazone successfully reduced macrophage necrosis in *M.tb* infection models [12], supporting the characterization of *M.tb*-induced macrophage necrosis as ferroptosis.

Another novel cell death pathway, cuproptosis, is driven by intracellular copper accumulation. Excess copper binds to acylated proteins in the tricarboxylic acid (TCA) cycle, causing protein aggregation, iron–sulfur cluster depletion, and cell death [13]. Additionally, copper catalyzes reactions that generate reactive oxygen species (ROS), further contributing to necrosis [14]. Thus, excessive copper levels lead to iron–sulfur cofactor destruction and ROS-mediated cell damage. Ferroptosis and cuproptosis have emerged as particularly relevant pathways in TB pathogenesis compared to other forms of cell death due to *M.tb*’s unique ability to manipulate iron and copper metabolism. Copper is essential for various biological functions, including mitochondrial respiration and antioxidant defense [15]. Emerging research suggests that copper may influence TB pathogenesis. For instance, G. Mohan observed decreased copper levels in TB patients after treatment [16], and Gnogbo Alexis Bahi et al. found a correlation between copper levels and multidrug-resistant TB [17]. These findings hint at copper’s potential role in TB pathogenesis, though its exact molecular mechanisms remain unclear. The specific effects of ferroptosis and cuproptosis on *M.tb* infection at the molecular and cellular levels remain incompletely understood. Clarifying these mechanisms could reveal insights into how these cell death pathways impact *M.tb* survival and replication and host immune responses. Furthermore, a better understanding of ferroptosis and cuproptosis in TB pathogenesis could inform new therapeutic strategies and biomarkers for disease severity and treatment response. This review aims to examine the roles of ferroptosis and cuproptosis in *M.tb* infection and explore therapeutic strategies targeting these pathways.

## 2. Ferroptosis: Mechanistic Insights, Key Features, and Its Role in Host–Pathogen Interactions During Tuberculosis

Ferroptosis is a form of iron-dependent cell death that is distinct from apoptosis, necroptosis, and autophagy due to its unique morphological, biochemical, and genetic characteristics (Figure 1). Unlike apoptosis, which is identified by nuclear fragmentation and chromatin condensation, and necrosis, which is marked by cell swelling and membrane rupture, ferroptosis is characterized by the accumulation of toxic lipid peroxides within cellular membranes, primarily polyunsaturated fatty acids (PUFAs) [18]. The process is driven by iron-catalyzed reactive oxygen species (ROS) that target and oxidize PUFAs, leading to lipid peroxidation and cellular damage. Unlike other forms of cell death, ferroptosis critically depends on the enzyme glutathione peroxidase 4 (GPX4), which detoxifies lipid peroxides. A deficiency or inhibition of GPX4 results in a buildup of lipid peroxides that destabilizes the plasma membrane, resulting in cell lysis and death [19]. Ferroptosis is, thus, a consequence of iron-induced lipid peroxidation combined with a diminished antioxidant defense mechanism, primarily due to reduced glutathione (GSH) levels, and subsequent GPX4 inhibition. Iron plays a central role in initiating this pathway through the Fenton reaction, which generates hydroxyl radicals capable of initiating lipid peroxidation and leading to widespread cellular injury. A decrease in cellular glutathione deactivates GPX4, further escalating lipid peroxide accumulation until the cell membrane ruptures [20]. Iron homeostasis—encompassing iron import, storage, and export—also influences ferroptosis susceptibility, as excessive free iron intensifies ROS production and lipid peroxidation, leading to increased cell vulnerability [21].

### 2.1. Ferroptosis in Infectious Diseases: TB and Host–Pathogen Interactions

In the context of infectious diseases, ferroptosis—a regulated form of cell death characterized by iron-dependent lipid peroxidation—has a dual impact by influencing both pathogen survival and host immune responses. For tuberculosis (TB), caused by *M.tb*, understanding the molecular interplay between ferroptosis and host–pathogen dynamics is critical for advancing therapeutic strategies. Despite its potential, the mechanisms by which *M.tb* manipulates host iron pathways and ferroptosis remain insufficiently explored. Recent research offers insights into these intricate processes, providing a foundation for detailed discussion.

#### 2.1.1. Host Iron Homeostasis and Ferroptosis in TB

Ferroptosis is tightly linked to iron metabolism. Host cells employ mechanisms such as “nutritional immunity” to limit iron availability to pathogens, thereby restricting their growth. This involves sequestering iron within ferritin, reducing extracellular iron via hepcidin upregulation, or exporting it through ferroportin. However, *M.tb* counters these strategies by actively modulating host iron pathways. For instance, *M.tb* secretes siderophores, like mycobactin and carboxymycobactin, to scavenge iron from the host environment, overcoming sequestration efforts [22,23]. This hijacking not only sustains *M.tb* replication but also dysregulates ferroptosis by disrupting host iron balance.

#### 2.1.2. Mechanisms of *M.tb* in Ferroptosis Modulation

The interplay between *M.tb* and host cell ferroptosis represents a critical axis in understanding the pathogen’s survival and virulence strategies. *M.tb* manipulates host iron homeostasis, antioxidant defenses, and lipid metabolism to create an environment conducive to its persistence and dissemination. By upregulating iron uptake and altering lipid peroxidation pathways, *M.tb* not only triggers ferroptotic cell death but also undermines the host immune response. This multifaceted modulation allows for *M.tb* to evade immune containment, disrupt granuloma integrity, and promote bacterial spread, highlighting ferroptosis as a potential target for therapeutic interventions.

- Iron Uptake and Redistribution: *M.tb* infection upregulates iron acquisition pathways in macrophages, including the expression of transferrin receptors and downregulation of ferroportin, which traps iron within cells. This iron accumulation promotes the Fenton reaction, generating reactive oxygen species (ROS) that can trigger lipid peroxidation—a hallmark of ferroptosis [10,24].
- Antioxidant Defense Manipulation: *M.tb* enhances its survival by interfering with host antioxidant defenses, particularly through the inhibition of glutathione peroxidase 4 (GPX4), a critical regulator of ferroptosis. Studies show that GPX4 activity is reduced in *M.tb*-infected macrophages, exacerbating lipid peroxidation and ferroptotic cell death. This dynamic supports the hypothesis that *M.tb* exploits ferroptosis to damage surrounding tissues, facilitating bacterial dissemination [25,26].
- Lipid Metabolism Alterations: *M.tb* infection alters host lipid metabolism by increasing polyunsaturated fatty acid (PUFA) production, which serves as substrates for lipid peroxidation during ferroptosis. Additionally, *M.tb*-derived factors can modulate host lipoxygenases, further driving ferroptotic pathways [27,28].
- Immune Evasion via Ferroptosis Induction: Excessive ferroptosis in macrophages compromises their ability to contain *M.tb*. By inducing lipid peroxidation and membrane damage, *M.tb* undermines the integrity of granulomas—organized immune structures essential for bacterial containment. This disruption aids *M.tb* in escaping immune surveillance and spreading to new tissues [10].

#### 2.1.3. Implications for TB Pathogenesis and Therapeutic Approaches

Balancing ferroptosis is crucial in TB. While inducing ferroptosis in *M.tb*-infected cells can restrict bacterial replication by depriving the pathogen of accessible iron, excessive ferroptosis can lead to detrimental inflammation and tissue damage. Therapeutic strategies targeting ferroptosis regulators, such as ferrostatins (ferroptosis inhibitors) or GPX4 activators, may help mitigate tissue damage while maintaining effective immune responses. Conversely, inducing controlled ferroptosis in infected macrophages through iron chelators or lipid peroxidation inducers might offer a novel means to limit *M.tb* replication [29,30]. The interplay between ferroptosis and *M.tb* pathogenesis is a complex but promising area for therapeutic intervention. By elucidating how *M.tb* manipulates host iron pathways and ferroptotic processes, future research can better inform targeted treatments. Recent studies highlight the importance of fine-tuning ferroptosis to balance pathogen control with preservation of host tissue integrity, paving the way for innovative approaches in TB management.

## 3. Copper-Induced Cell Death: Role of Copper in Cellular Metabolism and Stress Response

**Figure 2:**
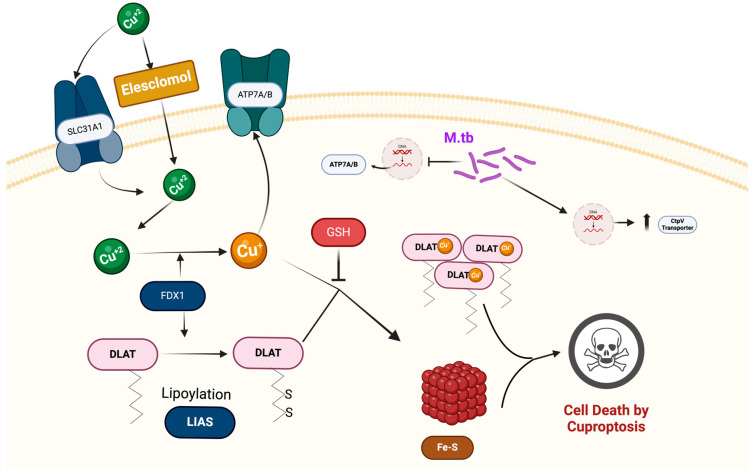
Cuproptosis occurs when there is an excess of copper (Cu^2+^) in the cell. Proteins, such as Cu^2+^ importers (SLC31A1) and exporters (ATP7B), play a role in controlling Cu^+^ levels within the cell and influence the cell’s sensitivity to copper-induced cell death. Elesclomol, a compound that binds to copper ions (Cu^2+^) outside the cell, helps transport these ions into the cell. Once inside, enzymes called reductases convert Cu^2+^ to Cu^+^ so it can enter the cell more easily. The buildup of Cu^2+^ within the cell leads to copper overload, which is largely caused by FDX1, a protein that triggers toxic stress in mitochondria. FDX1 also reduces Cu^2+^ to Cu^+^ and enhances the binding and aggregation of enzymes involved in the mitochondrial TCA cycle, particularly DLAT. At the same time, FDX1 destabilizes Fe–S cluster proteins. The presence of the Cu^2+^ chelator GSH, which contains thiol groups, can block the process of cuproptosis. Figure created with BioRender.com, accessed on 22 October 2024). DLAT: Dihydrolipoamide Acetyltransferase. FDX1: Ferredoxin 1. GSH: Glutathione. SLC31A1: Solute Carrier Family 31 Member 1 (also known as CTR1, Copper Transporter 1). LIAS: Lipoic Acid Synthetase. ATP7A/B: ATPase Copper Transporting Alpha/Beta (ATP7A and ATP7B are copper-transporting ATPases.

Copper is vital for cellular processes, including oxidative phosphorylation and redox reactions; however, dysregulated copper levels disrupt these essential functions, leading to cellular damage and cuproptosis (Figure 2). Unlike other forms of copper-mediated toxicity, cuproptosis does not primarily rely on oxidative stress but instead disrupts mitochondrial metabolic pathways through copper accumulation, leading to mitochondrial damage and subsequent cell death. Copper imbalances have been associated with multiple pathologies, including neurodegenerative diseases, cancer, and infectious diseases such as TB. Thus, maintaining cellular copper balance is critical for metabolic regulation and stress adaptation [31].

### 3.1. Cuproptosis: Mechanistic Features and Cellular Impact

Cuproptosis is a newly identified form of programmed cell death distinct from apoptosis, necrosis, and ferroptosis, driven by cellular copper accumulation that disrupts metabolic homeostasis and triggers cell death. While copper is indispensable for numerous enzymatic functions, disturbances in copper homeostasis induce cuproptosis. This process is regulated by copper-transporting proteins, such as ATP7B and CTR1, which facilitate copper import, distribution, and detoxification. The precise threshold of copper required to initiate cuproptosis is currently under investigation and varies depending on cellular copper transport mechanisms and the cell’s redox state [31].

A distinguishing feature of cuproptosis is its link to mitochondrial metabolism, as copper ions accumulate within mitochondria, binding to enzymes in the tricarboxylic acid (TCA) cycle and leading to protein aggregation and mitochondrial dysfunction [32]. Unlike other forms of cell death, which typically involve ROS generation or DNA fragmentation, cuproptosis results from copper-induced metabolic disruption. This unique pathway underscores its potential significance in diseases associated with mitochondrial dysfunction and copper dysregulation, such as neurodegenerative disorders and certain cancers [33]. Mechanistically, cuproptosis initiates as copper binds to lipoylated proteins like pyruvate dehydrogenase (PDH) within the TCA cycle, causing aggregation of metabolic enzymes and subsequently impairing mitochondrial function, ultimately resulting in cell death [32].

### 3.2. Cuproptosis in Infectious Diseases: TB and the Host–Pathogen Interface

In infectious diseases, including tuberculosis (TB), cuproptosis (copper-induced cell death) has emerged as a potential mechanism by which hosts can counteract pathogens. The intracellular bacterium *M.tb*, responsible for TB, encounters elevated copper levels within macrophage phagosomes as part of the host’s antimicrobial defense. Copper exhibits potent antimicrobial properties through its ability to disrupt bacterial proteins and generate reactive oxygen species (ROS) that lead to oxidative stress and cellular damage. Recent studies have identified that copper’s toxic effects in *M.tb* are mediated through interference with iron–sulfur cluster-containing proteins, which are critical for bacterial respiration and redox balance. This mechanism aligns with the cellular targets implicated in cuproptosis, suggesting that host cells may exploit copper toxicity to enhance pathogen clearance [34].

Despite these pressures, *M.tb* has evolved sophisticated mechanisms to evade copper-induced cell death. One key strategy involves copper efflux pumps, such as the P-type ATPase transporter (CtpV), which actively exports excess copper from the bacterial cytoplasm. A study by Festa et al. (2020) demonstrated that the ctpV gene is crucial for *M.tb* survival under copper stress, with its deletion resulting in increased bacterial susceptibility to copper toxicity and reduced virulence in a mouse model of TB [35]. Additionally, *M.tb* utilizes metallochaperones, such as RicR, to reprogram metal ion homeostasis and mitigate oxidative damage induced by copper. This regulatory system tightly controls the expression of copper-binding proteins and enzymes, thereby limiting intracellular copper accumulation and preserving metabolic stability [36].

Furthermore, *M.tb* may manipulate host immune pathways to reduce copper-mediated stress. For instance, it has been observed that *M.tb*-infected macrophages exhibit altered expression of copper transporters, such as CTR1 and ATP7A, potentially to sequester or redistribute copper away from sites of bacterial containment. Recent findings suggest that this immune evasion strategy could impair phagosomal copper loading, thereby dampening the antimicrobial efficacy of macrophages [37].

In the context of TB pathogenesis, the dual role of copper as both an antimicrobial agent and a regulator of immune signaling underscores its therapeutic potential. Inducing cuproptosis in macrophages or disrupting *M.tb*’s copper resistance pathways could amplify copper toxicity and limit bacterial replication. Studies exploring copper ionophores, compounds that increase intracellular copper levels, have shown promise in preclinical models by selectively targeting bacterial defenses while sparing host cells. For instance, a study by Djoko et al. (2020) demonstrated that combining copper ionophores with first-line TB antibiotics enhanced bacterial killing, suggesting a synergistic therapeutic approach [38].

Despite these advancements, the molecular mechanisms by which *M.tb* modulates host copper pathways remain incompletely understood. Investigating the crosstalk between copper metabolism, oxidative stress responses, and immune signaling in *M.tb*-infected cells is crucial for elucidating novel therapeutic strategies. Future studies should focus on the identification of host and bacterial factors that influence copper dynamics, the role of copper-binding proteins in immune regulation, and the integration of copper-based therapies in TB management.

## 4. Synergistic Interactions of Ferroptosis and Cuproptosis in Tuberculosis Pathogenesis

### 4.1. Combined Effects on Host Cells

#### 4.1.1. Impact on Macrophage Function

A diverse array of programmed cellular mechanisms exists, such as apoptosis, necrosis, and autophagy, to regulate cell death; however, ferroptosis and cuproptosis are unique forms of bacterial eradication that researchers are continuously exploring. Ferroptosis enhances macrophage bactericidal activity by accumulating lipid peroxides and oxidative damage in an iron-dependent manner, while cuproptosis capitalizes on the toxicity of copper to control macrophage stress and pathogen survival [39,40]. Together, these forms of cell death regulate macrophage function and enhance bacterial clearance to balance the control of infections and prevent excessive tissue damage.

The essential hallmarks of ferroptosis include free iron, mitochondrial superoxide formation, and lipid peroxidation in order to control bacterial load [39]. Although ferrous iron is an essential micronutrient for living organisms, its high reactivity can trigger the generation of reactive oxygen species (ROS) via the Fenton reaction [41]. These hydrogen peroxides induce phospholipid destabilization to form lipid peroxides, leading to the iron-dependent form of cell death called ferroptosis [42]. Specifically, it prevents host cell injury and effectively clears infections through the selective oxidation of membrane arachidonic acid-phosphatidylethanolamines (AA-PE) by 15-lipoxygenases [39]. Under steady-state conditions, these lipid peroxides are quickly neutralized by glutathione (GSH) and glutathione peroxidase isozymes (Gpx4) via glutathione oxidation [42]. However, in the face of excessive iron levels and generation of free radicals, Gpx4 expression becomes compromised, and lipid peroxides accumulate unchecked, providing one possibility to generate pro-inflammatory macrophages and necrotic cell death [42].

In contrast, copper plays a crucial role in immune responses by serving as a micronutrient in catalytic processes and as a structural cofactor for metal-dependent enzymes [43]. Cuproptosis operates through a different mechanism in which the accumulation of copper (Cu) ions disrupts mitochondrial dysfunction and macrophage metabolism [40]. Through the interaction with lipid components of the tricarboxylic acid (TCA) cycle, copper binds to mitochondrial enzymes, such as intracellular dihydrolipoamide S-acetyltransferase (DLAT) [40]. Copper subsequently induces the agglomeration of lipoylated proteins and loss of iron–sulfur clusters, resulting in copper homeostasis dysregulation [42,44]. Whether it be oxidative stress, endoplasmic reticulum stress, nucleolar stress, or proteasome inhibition, copper-dependent death significantly impacts mitochondrial respiration and cellular destruction [44].

Recent studies have revealed that increased ROS production and mitochondrial damage during ferroptosis can also activate pro-inflammatory signaling pathways in macrophages, such as the NF-κB and MAPK pathways, which further amplify immune responses [45]. This inflammatory response plays a critical role in the clearance of *M.tb* as well as in the modulation of adaptive immunity. Additionally, macrophages undergoing ferroptosis can release damage-associated molecular patterns (DAMPs), such as high-mobility group box 1 (HMGB1), which act as alarmins to recruit and activate other immune cells, including dendritic cells and T cells [46]. These DAMPs promote the initiation of an adaptive immune response, further enhancing bacterial clearance.

In contrast, copper plays a crucial role in immune responses by serving as a micronutrient in catalytic processes and as a structural cofactor for metal-dependent enzymes [43]. Cuproptosis, by regulating copper homeostasis in macrophages, also influences the release of pro-inflammatory cytokines, which play a pivotal role in the innate immune response to *M.tb* [47]. High copper concentrations can induce IL-1β and TNF-α secretion through the NF-κB pathway, further exacerbating immune responses that attempt to clear the pathogen. Additionally, the activation of copper-induced stress pathways can result in the recruitment of more immune cells to the site of infection, including neutrophils and dendritic cells, thus contributing to the overall inflammatory response [47]. However, excessive copper accumulation can also lead to immune suppression if it persists, potentially contributing to chronic infection and immune evasion by *M.tb* [48].

#### 4.1.2. Influence of Ferroptosis and Cuproptosis on Tuberculosis Immunity

The interplay between ferroptosis and immune signaling pathways has significant implications for the pathogenesis of infection and the development of various diseases. Numerous studies have reported that activating inflammatory pathways is a driving force for ferroptosis, including the Janus kinase-signal transducer and activator of transcription (JAK-STAT), nuclear factor-κB (NF-κB), inflammasome, cyclic GMP-AMP synthase-stimulator of IFN genes (cGAS-STING), and mitogen-activated protein kinase (MAPK) pathways [49]. In these pathways, increased cytokine production, such as interleukin (IL)-1, IL-6, IL-22, and interferon-α (IFN-α), upregulates hepcidin expression and enhances ROS levels [50]. Whether it be mediating downstream gene transcription, activating pro-caspases, or inducing oxidative DNA damage, these pathways deplete antioxidant activity and promote sequestration of extracellular iron [49,50]. It is clear that these pathways play a critical role in signal transduction and pro-inflammatory gene expression in order to disrupt iron metabolism and favor ferroptosis outcomes [49].

Ferroptosis also influences immune cell infiltration, which is critical for the resolution of infections. The release of DAMPs from ferroptotic macrophages can attract immune cells, including T cells and neutrophils, to the site of infection. This immune cell recruitment is essential for the resolution of infection, but in the case of *M.tb*, excessive inflammation can lead to tissue damage and the formation of granulomas, a hallmark of tuberculosis [51]. Additionally, the oxidative stress induced by ferroptosis can activate the cGAS-STING pathway, which is known to be involved in the detection of cytosolic DNA and subsequent activation of the innate immune response, further enhancing immune activation in the context of *M.tb* infection [52].

Similarly, cuproptosis has an intricate relationship with signaling pathways in order to impact cellular behavior and enhance inflammatory activity. Copper incorporates its properties into the tumor microenvironment (TME), where it enhances immune recognition of cancer cells through proliferation, angiogenesis, and metastasis [53]. Specifically, Cu strongly exerts influence on ligand-independent receptor tyrosine kinase (RTK) signaling, leading to the phosphorylation of extracellular regulated protein kinases (ERK) and agammaglobulinemia tyrosine kinase (ATK) [53,54]. Activation by Cu ions further catalyzes the redistribution of forkhead box (Fox) O1a and FoxO4, in turn promoting cancer cell migration and proliferation [54]. The binding between Cu and mitogen-activated protein kinase 1 (MEK1) also promotes ERK 1/2 phosphorylation, which then enhances downstream c-Jun N-terminal kinase (JNK) activity [54]. These complex mechanisms are essential to regulating tumor growth, offering insights into potential therapeutic targets for cancer and immune-related diseases.

In the context of *M.tb* infection, copper-induced immune activation also plays a crucial role in regulating macrophage responses. Studies have shown that copper accumulation in macrophages can enhance the bactericidal activity of these immune cells by stimulating the production of pro-inflammatory cytokines and ROS [47]. However, excessive copper levels can impair macrophage function and lead to the suppression of adaptive immunity. The balance between copper toxicity and immune activation may be key to controlling *M.tb* infection, as *M.tb* is known to manipulate copper metabolism to evade immune surveillance [48].

### 4.2. Pathogenesis Modulation by Ferroptosis and Cuproptosis in TB

#### 4.2.1. Bacterial Survival and Replication

Although ferroptosis and cuproptosis were found to underlie the pathophysiology of cardiomyopathy, acute renal failure, or neurodegeneration, their effects on *M.tb* growth are remarkable [39]. After the inhalation of aerosolized droplets, active *M.tb* relies on its ability to spread between infected alveolar macrophages within a tissue and to move between different organs in disseminated disease [42]. By inducing necrotic cell death via macrophages, this intracellular pathogen significantly increases the levels of free iron and lipid peroxides while simultaneously decreasing the levels of GSH and Gpx4, similar to the mechanisms of ferroptosis [50]. The uncontrolled production of ROS results in disruption of the plasma membrane and decreased host resistance, facilitating mycobacterial spread throughout target tissues [42]. What was once an effective modality for host cells to control pathogens has now become a weapon that *M.tb* manipulates to promote disease development.

Recent studies have highlighted that ferroptosis is involved in *M.tb*-induced tissue damage and disease progression. The lipid peroxidation induced by ferroptosis can further increase the ROS burden in infected tissues, exacerbating inflammation and leading to tissue necrosis [55]. Moreover, the release of DAMPs from ferroptotic cells can activate the STING pathway, which plays a crucial role in the host’s immune response to *M.tb* infection [52].

Research has newly revealed a multifaceted relationship between copper metabolism and *M.tb* survival. It is evident that cuproptosis drives the toxic accumulation of Cu in macrophage phagolysosomes as part of the host’s innate immune response to minimize bacterial growth [56]. However, *M.tb* has developed advanced strategies to counteract copper toxicity by exploiting its antimicrobial properties and increasing bacterial resistance to oxidative stress [56,57]. Through copper tolerance mechanisms, it encourages the binding of the mycobacterial chaperone GroEL1 to copper ions in efforts to reduce their toxic effects [57]. Moreover, the loss of the copper-sensitive operon repressor (CsoR) leads to decreased transcriptional regulation and increased survival under copper stress [58]. These evolved mechanisms highlight how both the pathogen and host manipulate copper metabolism, which is crucial for the survival and virulence of *M.tb*-infected macrophages within the host. With continuous research in this field, scientists can further delve into experimental medicine and explore alternative therapies to mitigate the pulmonary necrosis caused by *M. tuberculosis*.

In line with these findings, cuproptosis is thought to play a dual role in *M.tb* infection. While copper overload in macrophages can trigger cuproptosis and help limit bacterial replication by inducing oxidative stress, *M.tb*’s ability to manipulate copper homeostasis enables it to avoid the bactericidal effects of copper accumulation [48]. The bacteria achieve this through copper resistance mechanisms, including the activation of copper efflux pumps and the expression of chaperones that bind excess copper [48].

#### 4.2.2. Host Resistance and Susceptibility

**Figure 3:**
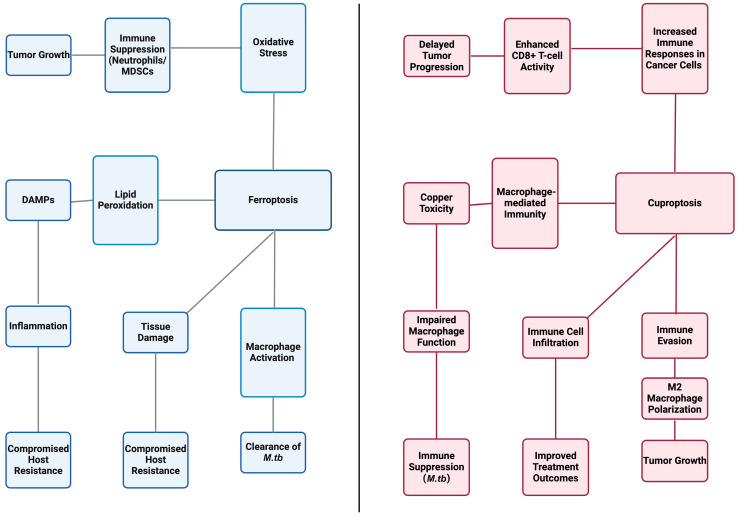
Host resistance and susceptibility. Ferroptosis and cuproptosis, two distinct cell death pathways, play crucial yet opposing roles in immunity and disease. Ferroptosis drives lipid peroxidation, triggering release of DAMPs, inflammation, and oxidative stress, leading to immune suppression and tumor growth while compromising host resistance. In contrast, cuproptosis enhances immune responses in cancer by promoting CD8+ T cell activity and immune cell infiltration, delaying tumor progression and improving treatment outcomes. However, cuproptosis may also facilitate immune evasion via M2 macrophage polarization, fostering tumor growth. In infections like tuberculosis (*M.tb*), ferroptosis activates macrophages for bacterial clearance but risks tissue damage and impaired host defense. Conversely, cuproptosis-induced copper toxicity can suppress macrophage function, weakening immunity against *M.tb*. Created in BioRender.com, accessed on 25 October 2024. MSDCs stands for Mitochondrial Small Molecule Drug Compounds.

Ferroptosis can modulate both innate and adaptive immune responses (Figure 3) in that it can lead to immune suppression in neutrophils and myeloid-derived suppressor cells (MDSCs) [59]. In the tumor microenvironment (TME), ferroptosis of MDSCs releases oxygenated lipids that inhibit T cell activity, thereby promoting tumor growth [59]. Conversely, inhibiting ferroptosis in these cells can enhance anti-tumor immunity and serve as an adjunct to immune checkpoint blockade therapies [59]. Not only does ferroptosis affect host defense mechanisms, but it can also accelerate the replication and dissemination of pathogens. Through lipid peroxidation and oxidative stress, ferroptosis activates the release of damage-associated molecular patterns (DAMPs), which further aggravates inflammation [60]. Similar to *M.tb*, certain bacteria and viruses induce these complex mechanisms to evade host surveillance, leading to disrupted tissue barriers, microbial entry, and exacerbated disease outcomes [60].

The influence on the TME is not solely limited to ferroptosis, as cuproptosis increases active immune responses in cancer cells, enhances infiltration of immune cells, and improves treatment outcomes with immune checkpoint blockers [61]. For example, the synergistic relationship between decreasing PD-L1 expression and downregulating the WNT signaling pathway enhances CD8+ T cell cytotoxicity, resulting in delayed progression of cancer cells [62]. However, cuproptosis plays a dual role and may also lead to immune evasion, complicating disease outcomes [61,63]. In the context of gliomas, high levels of cuproptosis are associated with increased macrophage infiltration, in turn contributing to a worse prognosis [63]. These macrophages often adopt an immunosuppressive M2 phenotype, which accelerates tumor growth and inhibits effective anti-tumor responses [63]. These unique effects of cuproptosis warrant further discussion on how to craft effective immunotherapies in order to enhance host defenses and mitigate disease severity. In the case of tuberculosis, ferroptosis and cuproptosis influence immune responses by modulating the activation and function of macrophages. While ferroptosis contributes to immune activation and the clearance of *M.tb*, excessive lipid peroxidation and inflammation can also lead to tissue damage, potentially compromising the host’s ability to resist infection. Similarly, cuproptosis contributes to macrophage-mediated immunity but may also lead to immune evasion by *M.tb*, as copper toxicity can impair macrophage function and promote immune suppression [48].

## 5. Innovative Therapeutic Opportunities in TB: Targeting Ferroptosis and Cuproptosis

### 5.1. Targeting Ferroptosis: Potential and Limitations in Tuberculosis Treatment

Over the past five years, specific therapeutic strategies have emerged to modulate ferroptosis, aiming to enhance the treatment of tuberculosis (TB) [Table 1]. A prominent approach involves the use of ferroptosis inducers, such as Erastin and RSL3, which function by elevating intracellular iron levels and reactive oxygen species (ROS) production. This increase leads to lipid peroxidation and subsequent cell death in infected macrophages. Preclinical studies have demonstrated that Erastin can be effectively administered at doses ranging from 5 to 10 μM to induce ferroptosis across various cell lines [64]. However, these findings remain primarily preclinical, as human studies evaluating the clinical applicability of ferroptosis inducers are currently lacking [Table 2]. Variability in patient-specific factors, such as immune responses and iron metabolism, may significantly affect therapeutic outcomes, underscoring the need for rigorous clinical trials to establish dosing, efficacy, and safety profiles. Similarly, RSL3 has been shown to trigger optimal ferroptosis at dosing levels of 0.25 to 0.5 μM in both in vitro and in vivo models, minimizing potential side effects [65]. The concurrent administration of RSL3 or Erastin alongside standard TB therapies, such as rifampin or isoniazid, holds promise for enhancing bactericidal effects by creating a highly oxidative environment that is detrimental to *Mycobacterium tuberculosis* [18].

Another promising therapeutic avenue involves the use of lipid peroxidation inhibitors, such as ferrostatin-1. Administered at doses between 1 and 5 μM, ferrostatin-1 has been shown to effectively regulate ferroptosis and protect uninfected cells from oxidative damage [66]. Despite its potential, the long-term use of ferrostatin-1 and its effects on other oxidative pathways remain underexplored. Extended inhibition of ROS could inadvertently compromise immune defenses, which rely on oxidative bursts to eliminate pathogens. Detailed in vivo studies are required to delineate these risks and assess safety in TB-specific contexts [67]. By modulating ROS levels within infected macrophages, this strategy may help mitigate collateral tissue damage while promoting efficient bacterial clearance.

Recent investigations have also focused on iron chelators, such as deferoxamine, which reduce the availability of iron stores and suppress ferroptosis under certain conditions. Clinically, deferoxamine has been utilized at doses ranging from 500 to 1000 mg/day for the management of iron-overload disorders; however, lower doses may suffice for modulating ferroptosis in TB patients [68]. The combination of deferoxamine with ferroptotic agents offers a balanced therapeutic strategy that leverages a well-established modulator alongside newer interventions [64]. However, prolonged iron depletion could inadvertently impair immune cell function or induce anemia, particularly in vulnerable TB patients. Mechanistic studies exploring the interplay between iron availability, ferroptosis, and host immunity are needed to address these potential concerns [69].

Furthermore, FIN56, a novel ferroptosis inducer that targets GPX4 degradation, has emerged as a potential therapeutic candidate. Preclinical trials indicate that effective concentrations for FIN56 range from 0.1 to 1 μM, enhancing lipid peroxidation and inducing ferroptosis [70]. Although promising, its application to TB remains theoretical, as studies directly linking FIN56 to *Mycobacterium tuberculosis* pathogenesis are absent. Most findings are based on generalized cell-line studies, highlighting a critical gap that warrants experimental validation in TB-specific models.

Despite the promise of ferroptosis-based therapies, a significant challenge remains in ensuring specificity for infected cells to avoid damaging healthy tissues. Similar to chemotherapy, precise dosing must be monitored to prevent excessive ROS accumulation and tissue inflammation, particularly in patients with anemia or iron-overload disorders. Furthermore, chronic modulation of ferroptosis may exacerbate pre-existing oxidative stress or comorbid conditions like cardiovascular diseases and neurodegenerative disorders. Advances in targeted drug delivery, such as nanoparticle systems, could help localize ferroptosis inducers to infected cells, reducing off-target effects and improving therapeutic safety [71]. Careful monitoring is especially crucial for individuals with comorbidities such as malignancies or bleeding disorders.

### 5.2. Therapeutic Exploration of Cuproptosis in Tuberculosis Management

Cuproptosis presents a novel therapeutic target for TB by leveraging the antimicrobial properties of copper. Copper ionophores, such as disulfiram and Elesclomol, facilitate the uptake of copper into macrophages, thereby promoting cell death in infected cells. Disulfiram has been utilized in clinical settings at doses of 250 to 500 mg/day and has shown promise for repurposing in TB treatment [72,73]. Similarly, Elesclomol, which has been administered at 5 to 10 mg/m^2^ in clinical trials for cancer, could be explored for inducing cuproptosis in TB-infected macrophages [72]. Nonetheless, direct evidence connecting cuproptosis mechanisms to *Mycobacterium tuberculosis* pathogenesis is absent. Most studies rely on bioinformatics and theoretical frameworks, emphasizing the need for empirical validation to quantify the role of cuproptosis in TB pathophysiology [74].

Recent studies indicate that combining copper ionophores with existing anti-TB drugs, such as Bedaquiline, produces synergistic effects. The combination of Elesclomol with Bedaquiline at a dosage of 50 mg/day has been shown to enhance bacterial clearance by leveraging the bactericidal properties of copper in conjunction with established therapies [72]. However, copper toxicity remains a major challenge. Excessive accumulation of copper can cause oxidative damage to non-infected tissues, particularly in the liver and kidneys, necessitating close monitoring of copper levels and careful dose optimization during therapy [67].

Copper chelators, such as Tetrathiomolybdate, which are administered at doses of 20 mg/day in patients with Wilson’s disease, could be beneficial in managing copper-induced toxicity among TB patients undergoing copper ionophoric therapy [75]. This strategy allows for the regulation of intracellular copper levels, ensuring sufficient copper accumulation to induce cuproptosis in infected cells while minimizing the risk of systemic toxicity. Moreover, recent advancements in nanoparticle delivery systems may provide a more targeted approach for modulating intracellular copper levels, potentially reducing toxicity [75]. Preclinical models have shown that nanoparticle formulations can enhance the delivery precision of copper ionophores while mitigating systemic side effects. Similar advancements in TB treatment could optimize the therapeutic index of cuproptosis-based strategies [76].

Additionally, ATN-224, a superoxide dismutase 1 (SOD1) inhibitor, has been investigated as a potential enhancer of cuproptosis. In preclinical studies, ATN-224 has been administered at doses of 50 to 100 mg/day, demonstrating promising results in increasing copper-mediated oxidative stress in infected cells while minimizing adverse effects on non-infected tissues [77]. Nevertheless, prolonged SOD1 inhibition may impair antioxidant defenses in non-target tissues, requiring further studies to evaluate its long-term safety and applicability in TB therapy [78].

However, copper toxicity remains a significant concern in cuproptosis-based therapies. Excessive copper accumulation can result in liver and kidney damage, necessitating close monitoring of copper levels during treatment. Furthermore, co-administered medications may require dose adjustments, as many of them are excreted renally or hepatically. Although copper chelators can help mitigate these risks, careful calibration and periodic adjustments of their dosing in conjunction with ionophores are essential [73]. Moreover, resistance mechanisms, such as copper efflux or metabolic adaptations in Mycobacterium tuberculosis, could compromise the efficacy of cuproptosis-inducing agents. Studies mimicking prolonged drug exposure are essential to explore the likelihood and mechanisms of such resistance [67].

Ongoing research is essential to assess the safety and efficacy of cuproptosis-targeted therapies across diverse patient populations. Innovations in nanoparticle formulations or controlled-release systems may facilitate more precise delivery methods, enhancing therapeutic outcomes while minimizing systemic toxicity [75].

## 6. Mitochondrial Dynamics in Macrophage Cell Death: Future Directions for Tuberculosis Therapies

Mitochondria play a critical role in regulating macrophage responses to *M.tb*, determining whether the cell undergoes apoptosis or necrosis. This balance is crucial, as apoptotic cell death is associated with bacterial containment, while necrosis often facilitates bacterial dissemination.

Recent studies have revealed that mitochondrial reactive oxygen species (ROS) production plays a key role in macrophage apoptosis during *M.tb* infection. Elevated ROS levels promote the release of cytochrome c, triggering caspase activation and apoptotic pathways that restrict bacterial replication [79]. However, *M.tb* has evolved mechanisms to manipulate mitochondrial function to avoid apoptosis. For example, the *M.tb* protein ESAT-6 induces mitochondrial damage, promoting necrotic cell death that benefits bacterial survival and dissemination [80]. In addition to ROS, mitochondrial dynamics—fusion and fission—are also implicated in macrophage death. Excessive mitochondrial fission, often driven by Drp1 activation, has been shown to disrupt mitochondrial membrane potential and drive necrosis during *M.tb* infection [81]. In contrast, studies have demonstrated that enhancing mitochondrial fusion protects macrophages against necrosis, preserving their ability to contain the bacteria [82]. Mitochondrial DNA (mtDNA) release and inflammasome activation further highlight the role of mitochondria in macrophage death. Upon mitochondrial damage, mtDNA is released into the cytosol, where it activates the NLRP3 inflammasome, leading to inflammatory cytokine release and pyroptosis [83]. This inflammasome-mediated cell death, although inflammatory, may aid in the clearance of infected cells under certain conditions [84].

Taken together, these findings underscore the dual role of mitochondria in macrophage death processes during *M.tb* infection, with potential implications for therapeutic targeting to enhance host defense mechanisms.

Future research should focus on advancing therapeutic strategies that target ferroptosis and cuproptosis, with an emphasis on specificity and minimizing systemic side effects. For instance, the development of nanoparticle-based drug delivery systems could be explored to localize ferroptosis inducers, such as Erastin and RSL3, directly to *M.tb*-infected macrophages. This approach would maximize the therapeutic impact while protecting healthy tissues from oxidative damage. Another promising direction is the combination of copper ionophores like Elesclomol with established anti-TB drugs such as Bedaquiline to exploit the synergistic effects of copper toxicity and bacterial clearance. Additionally, integrating iron chelators such as deferoxamine with ferroptosis modulators presents an opportunity to balance pathogen suppression with controlled immune responses, reducing tissue damage. Future studies should also investigate how *M.tb* exploits these cell death pathways to evade immune defenses, potentially uncovering new molecular targets for therapeutic intervention. Furthermore, identifying biomarkers associated with ferroptosis and cuproptosis could enhance our ability to monitor disease progression and tailor treatments to individual patients. These directions hold significant potential for developing innovative and more effective TB management strategies.

## 7. Methods

Data were collected based on a web search of recent scientific peer-reviewed journals. Key words used in the data search using PubMed as a search engine and its database include ferroptosis, cuproptosis, *M.tb*, oxidative stress, macrophages, cell death, host resistance and susceptibility, and therapeutics.

## 8. Conclusions

Tuberculosis (TB) caused by *M.tb* remains a global health crisis, with over 10 million people affected annually. Despite advancements in treatment, *M.tb* has developed mechanisms to evade host immune responses, complicating efforts to eradicate the disease. Two emerging cell death pathways, ferroptosis and cuproptosis, have been linked to TB pathogenesis. Ferroptosis, an iron-dependent form of cell death, is driven by lipid peroxidation and reactive oxygen species (ROS) accumulation. This process can limit *M.tb* replication by depleting intracellular iron and inducing macrophage necrosis. However, excessive ferroptosis may lead to tissue damage and aid bacterial dissemination. Cuproptosis, triggered by copper accumulation, disrupts mitochondrial metabolism, leading to protein aggregation and cell death. *M.tb* exploits both iron and copper metabolism to survive within macrophages, manipulating these processes to resist oxidative stress and immune responses. This review examines the roles of ferroptosis and cuproptosis in TB, discussing how *M.tb* manipulates these pathways for survival. While therapeutic strategies targeting these processes, such as ferroptosis inducers (Erastin, RSL3) and inhibitors (Ferrostatin-1) and copper ionophores (Disulfiram, Elesclomol) and chelators, show promise, the limited understanding of these pathways and potential off-target effects remain significant challenges. Further exploration of these pathways may provide insights into the development of targeted therapies aimed at controlling *M.tb* infection while minimizing host tissue damage. By elucidating the complex interactions between ferroptosis, cuproptosis, and TB, future therapies could better address bacterial resistance and improve clinical outcomes.

## Figures and Tables

**Table 1:** Summary of therapeutics.

Therapeutic Strategy	Compound	Dose	Mechanism	Potential Challenges
Ferroptosis Inducers	Erastin	5–10 μM	Increases iron and ROS for lipid peroxidation and cell death	Ensuring specificity to infected cells to prevent healthy cell damage
	RSL3	0.25–0.5 μM	Triggers ferroptosis in infected cells	Dose monitoring to avoid excessive ROS and tissue inflammation
	FIN56	0.1–1 μM	Enhances lipid peroxidation by targeting GPX4	Long-term toxicity with chronic iron manipulation
Lipid Peroxidation Inhibitors	Ferrostatin-1	1–5 μM	Balances ROS to protect uninfected cells	Potential systemic oxidative stress
Iron Chelators	Deferoxamine	500–1000 mg/day (lower in TB)	Reduces iron to control ROS and ferroptosis	Risk of iron deficiency or worsening anemia
Cuproptosis Inducers	Disulfiram	250–500 mg/day	Enhances copper uptake for cell death in infected cells	Copper toxicity risks
	Elesclomol	5–10 mg/m^2^	Facilitates copper ion uptake	Liver and kidney toxicity from excess copper
Copper Chelators	Tetrathiomolybdate	20 mg/day	Manages copper toxicity	Need for dose adjustment and monitoring
SOD1 Inhibitor	ATN-224	50–100 mg/day	Enhances oxidative stress in infected cells	Monitoring to minimize non-infected

RSL3: Ras-selective lethal small molecule 3. FIN56: Ferroptosis Inducer 56. ATN-224: A Tetrahydroxy Naphthalene 224 (specific compound identifier for a copper chelator). SOD1: Superoxide Dismutase 1. ROS: Reactive Oxygen Species.

**Table 2:** Chemical properties and structure of therapeutics.

Compound Name	Chemical Structure (IUPAC Name)	Type	Clinical Usage
Erastin	2-[1-[4-[2-(Dimethylamino)ethoxy]phenyl]ethylidene]indolin-3-one	Small molecule, ferroptosis inducer	Not used clinically
RSL3	1S,3R-RSL3: [(1S,3R)-2-chloro-3-[[(2,4-dichlorobenzyl)sulfanyl]methyl]cyclohexyl]methyl sulfide	Small molecule, ferroptosis inducer	Not used clinically
FIN56	*N*-[4-[[4-(1,3-Benzothiazol-2-yl)piperidin-1-yl]methyl]phenyl]quinolin-4-amine	Small molecule targeting GPX4	Not used clinically
Ferrostatin-1	3-[4-(Phenylamino)cyclohexyl]propanoic acid	Small molecule, lipid peroxidation inhibitor	Not used clinically
Deferoxamine	*N*-[5-[[4-[5-(Acetylhydroxyamino)pentylamino]-4-oxobutanoyl]amino]pentyl]-*N*-hydroxyacetamide	Iron chelator	Used clinically for iron-overload conditions
Disulfiram	*N*,*N*-Bis(diethylthiocarbamoyl)disulfide	Alcohol deterrent and cuproptosis inducer	Used clinically for alcohol dependency
Elesclomol	2-(4-Chlorophenyl)-1-[3-(dimethylamino)propyl]-1,3-dihydroimidazol-3-one	Anticancer agent and cuproptosis inducer	Investigated in clinical trials; not widely used clinically
Tetrathiomolybdate	MoS_4_^2−^	Copper chelator	Investigated in clinical trials; limited clinical use
ATN-224	Ammonium tetrathiomolybdate	Small molecule, SOD1 inhibitor	Investigated in clinical trials; not used clinically

SOD1: Superoxide Dismutase 1. GPX4: Glutathione Peroxidase 4.

## Data Availability

Data are available in the references.
